# L-kynurenine induces NK cell loss in gastric cancer microenvironment via promoting ferroptosis

**DOI:** 10.1186/s13046-023-02629-w

**Published:** 2023-03-01

**Authors:** Jian-Xin Cui, Xian-Hui Xu, Tao He, Jia-Jia Liu, Tian-Yu Xie, Wen Tian, Jun-Yan Liu

**Affiliations:** 1 Department of General Surgery, The First Medical Center, Chinese PLA General Hospital, Beijing, 100853 China; 2 Department of Emergency, No. 971 Hospital of PLAN, Qingdao, 266071 Shandong Province China; 3 Department of General Surgery and Center of Minimal Invasive Gastrointestinal Surgery, Southwest Hospital, Third Military Medical University (Army Medical University), Chongqing, 400038 China

**Keywords:** NK cell, Gastric cancer, Tumor microenvironment, L-kynurenine, Ferroptosis

## Abstract

**Background:**

Natural killer (NK) cells play a major role in body’s fighting against various types of cancers. Their infiltration in the tumor microenvironment (TME) of gastric cancer (GC) are significantly decreased, which has been reported as a robust prognostic marker. However, the causes leading to NK cells loss in GC TME remains poorly understood.

**Methods:**

We constructed a non-contact co-culturing system and humanized xenograft tumor mice model to detect the influence of GC microenvironment on NK-92 or primary human NK cells viability by flow cytometry. Then through using the specific inhibitors for different types of cell death and examining the surrogate markers, we confirmed ferroptosis in NK cells. Inspired by the accidental discoveries, we constructed a NK-92 cell strain with high expression of GPX4 and treated the humanized xenograft tumor mice model with the NK-92 cells.

**Results:**

We found L-KYN, mainly generated through indoleamine 2, 3-dioxygenase (IDO) from GC cells, impaired NK cells viability in TME. Further analysis revealed L-KYN induced ferroptosis in NK cells via an AHR-independent way. Moreover, we found NK cells with higher GPX4 expression showed resistance to L-KYN induced ferroptosis. Based on this, we generated GPX4 over-expressed NK-92 cells, and found these cells showed therapeutic potential towards GC.

**Conclusions:**

Our study revealed a novel mechanism to explain the decline of NK cell number in GC TME. Notably, we also developed a potential immunotherapy strategy, which might be beneficial in clinical treatment in the future.

**Supplementary Information:**

The online version contains supplementary material available at 10.1186/s13046-023-02629-w.

## Introduction

The global incidence of gastric cancer (GC) ranks among the top five of all types of tumors [1]. As it is usually diagnosed at the advanced stage, this makes gastric cancer the third most cause of tumor-related deaths [2]. Although there are great advances in surgical procedures or other therapies, the overall 5-year survival rate of GC still remains under 30% in most countries [3]. The interactions between tumor and immune cells in the local microenvironment are believed to be involved in the growth, metastasis and invasion of GC [2]. Moreover, the complexity of tumor microenvironment (TME), which mainly consists of neoplastic cells, stromal cells, immune cells, soluble factors and extracellular matrix, severely diminishes the efficacy of anti-tumor immunity, thus restraining the effects of various immunotherapies developed during the past decades. For example, the PD-1/PD-L1 treatment have shown outstanding effects on several types of cancer, but the objective response rate in GC was only at 15% [4]. Hence, clarifying the mechanisms involved in TME regulation on anti-tumor immunity might provide potential targets.

Natural killer (NK) cells are an integral component of the innate immune system, which undertake the immune surveillance against tumors [5]. There are mounting evidence suggesting that the improvement of NK infiltration or function in tumors is obviously beneficial for patient survival [6]. NK cells recognize the targets through the biased signals transmitted from active or inhibitory receptors. After activation, they can directly lyse the targets by generating cytotoxic molecules, such as perforin and granzyme B. Besides, NK cells also secret amounts of immune regulators, especially IFN-γ, to enhance the overall anti-tumor activity [5]. Therefore, NK-based immunotherapies have attracted great attentions these years [7].

NK cells are reported to be tightly associated with the occurrence and development of GC. Their abundance in GC TME is dramatically reduced, which is considered as one of the most robust prognostic markers to GC [8]. A multivariate Cox analysis was performed to show that the intra-tumoral NK cell density was positively related with the overall and disease-free survival of GC patients [9]. Notably, most of the NK cells in GC TME exhibited a normal cytotoxic phenotype and secreted normal levels of cytokines [9]. Thus, restoring the number of NK cells in TME is of great significance for GC clinical treatment. However, the reasons leading to NK cells decrease needs to be further clarified.

Recently, several studies have revealed that the accumulation of L-kynurenine (L-KYN) mediate immunosuppressive effects in TME [10]. L-KYN is a major component of the tryptophan metabolites, in which process indoleamine 2,3-dioxygenase (IDO) acts as the rate-limiting enzyme [11]. IDO is generally absent or inactivated in immune cells, except the activated macrophages or DC cells [12–14], but IDO has been reported to be excessively expressed in GC cells, resulting in higher generation of L-KYN [15]. L-KYN is considered to exert immunosuppressive effects through the aryl hydrocarbon receptor (AHR), mainly leading to suppressed proliferation of effector T cells and enhanced generation of T-reg cells [16–18]. However, the influence of L-KYN on NK cells remains largely unknown.

In the present study, in virtue of the co-culture system in vitro and humanized mice model in vivo, we proved that L-KYN, which was generated by IDO from GC cells, induced NK cells ferroptosis in an AHR-independent way, leading to their loss in GC TME. Interestingly, we found some primary human NK cells with higher expression of GPX4 were resistant to L-KYN-induced cell death. Inspired by this phenomenon, we constructed the GPX4-overexpressed NK-92 cells and found they showed therapeutic effects towards GC in the humanized mice. Hopefully, our study could provide novel strategy to develop NK-based immunotherapy for GC treatment.

## Materials and methods

### Mice

The 8-week-old female hIL-15 NOG (NOD.Cg-PrkdcscidIl2rgtm1SugTg (CMV-IL2/IL1 5)1-1Jic/JicCrl) mice were purchased from Charles River Laboratories. This mouse strain expresses endogenous human IL-15 cytokine, which can successfully reconstruct and maintain the peripheral blood-derived human NK cells (PB-hNK) in vivo for as long as 3 months [19]. The mice were bred in the special pathogen-free facility with free access to water and food, 12-h light/dark cycle, periodic air changes and 25 °C constant temperature at the Experimental Animal Center of the Chinese PLA General Hospital. All the animal experiments were approved by the Animal Ethics Committee of the Chinese PLA General Hospital (#20190112).

### Cell lines

NK-92 cells were commercially purchased from the Procell Life Science&Technology Co.,Ltd. with authentication. GES-1, SGC-7901 were maintained in our own lab and had been authenticated by STR profiling. The complete medium to maintain the cell lines were as follows: GES-1, SGC-7901 and K562: RPMI-1640 medium + 10% fetal bovine serum (FBS) + 1% penicillin / streptomycin; NK-92: MEMα+ 0.2 mM inositol+ 0.1 mM β-mercaptoethanol+ 0.02 mM folic acid+ 100-200 U/mL recombinant IL-2 + 12.5% horse serum+ 12.5% FBS + 1% penicillin / streptomycin. All the cultured cells were regularly tested and confirmed negative for mycoplasma contamination.

### Measurement of L-KYN concentration

As our co-culture assays were performed in the presence of NK-92 complete medium, we measured the concentration of L-KYN when GES-1 or SGC-7901 cells were cultured with this medium, which showed no influence on the viability and proliferation of these cells (data not shown). 5 × 10^5^ GES-1 or SGC-7901 cells were seeded in the plate with 1 ml NK-92 complete medium. Five milligram per milliliter tryptophan (half of the content in MEMα medium) was supplemented into the culture medium every 24 h after beginning. Then the concentration of L-KYN in the culture supernatant was measured with the Kynurenine ELISA Kit (abcam), following the manufacture’s instruction.

### Non-contract co-culture system

The non-contract co-culture assays were performed in the 12-well plate (Corning) and 0.4 μm Transwell® membrane inserts (Corning). 5 × 10^5^ GES-1 or SGC-7901 cells were seeded in the lower chamber with 500 μL NK-92 complete medium and the same number of NK-92 cells were seeded in the upper chamber with 500 μl medium. Then the suspended NK-92 cells were collected for the subsequent analysis.

### Reagents and cell treatment

**Fig. 1:**
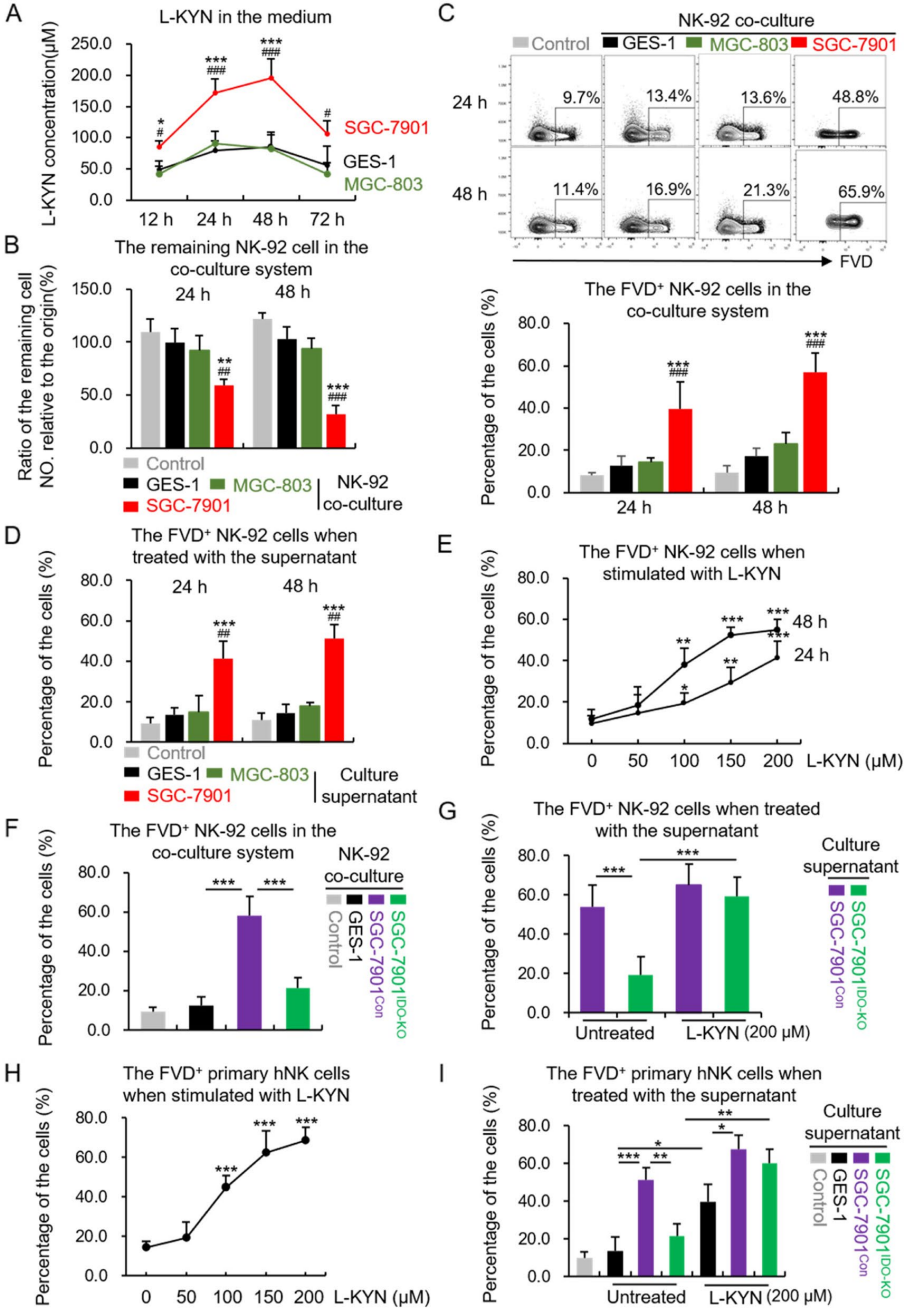
IDO produced L-KYN from GC cells impairs NK viability in vitro. **A** The concentration of L-KYN in the culture supernatant of SGC-7901, MGC-803 or GES-1 cells measured by ELISA. **B** The ratio of the remaining NK-92 cell number relative to the originally seeded cell number in the non-contract co-culture system with GES-1, MGC-803 or SGC-7901 cells. The control group referred to that the NK-92 cells were seeded into the co-culture wells alone. **C** The flow cytometric analysis for the proportion of FVD^+^ NK-92 cells in the co-culture system. The pictures above showed the representative results. **D** The flow cytometric results that showed the proportion of FVD^+^ NK-92 cells when stimulated with the culture supernatant of GES-1, MGC-803 or SGC-7901 cells. The control group referred to the medium without culturing with cells. **E** The proportion of FVD^+^ NK-92 cells when treated with gradient concentrations of L-KYN for 24 or 48 h. **F and G** The proportion of FVD^+^ NK-92 cells when co-cultured with GES-1, SGC-7901^Con^, SGC-7901^IDO-KO^ (**F**), or treated with the culture supernatant of SGC-7901^Con^ or SGC-7901^IDO-KO^ (**G**) for 48 h. The control group in (**F**) indicated the NK-92 cells were only treated with medium. **H** and **I** The proportion of FVD^+^ primary human NK (hNK) cells when treated with gradient concentrations of L-KYN for 48 h (**H**) or the culture supernatant from GES-1, SGC-7901^Con^ and SGC-7901^IDO-KO^ cells (**I**). All the results were replicated in 3 (**H** and **I**) or 4 (**A - G**) independent experiments. * refers to the *p*-value of group SGC-7901 vs GES-1 and # refers to the group SGC-7901 vs MGC-803 (**A - D**). * *p* < 0.05, ** *p* < 0.01, *** *p* < 0.001, # *p* < 0.05, ## *p* < 0.01, ### *p* < 0.001

L-Kynurenine (L-KYN), CH-223191, ferrostatin-1 (Fer-1), necrostatin-1 (Nec-1), Z-VAD-FMK (Z-VAD), VX-765 and GPX4-IN-3 were all purchased from MedChemExpress. We conducted preliminary experiments to determine the effective concentration and safety of each inhibitor on NK cells. For the culture supernatant treatment assays, the GES-1 or SGC-7901 cells were changed to be cultured with NK-92 complete culture medium for 24 h, which contained the highest concentration of L-KYN as determined in Fig. 1A. The supernatants were collected and mixed with 20% fresh medium, which were then used to treat NK cells for 24 or 48 h.

### Flow cytometric analysis

Fixable Viability Dye (FVD) staining: The Fixable Viability Dye eFluor™ 506 was commercially purchased from Invitrogen™. The collected NK-92 cells were re-suspended with 500 μl FVD staining solution (1:800 diluted in PBS) and incubated at room temperature for 15 min in the dark. The staining was stopped by washing cells with 2.5 ml PBS (containing 10% FBS). Then the cells were immediately analyzed by a BD FACSCelesta cytometer or fixed for intracellular antibodies staining.

Ki-67 and GPX4 (Glutathione Peroxidase 4) staining: The APC anti-Human Ki-67 antibody and APC Mouse IgG1, κ Isotype Control antibody were purchased from Biolegend. The anti-Glutathione Peroxidase 4 antibody (primary antibody), Goat anti-Rabbit IgG H&L (Alexa Fluor® 405) (fluorescence secondary antibody) and Rabbit IgG, monoclonal [EPR25A] - Isotype Control were purchased from Abcam. Following the FVD staining, the intracellular staining of Ki-67, GPX4 or the corresponding isotype controls were performed with the Foxp3 / Transcription Factor Staining Buffer Set (eBioscience™) following the manufacturer’s instructions.

Intra-tumoral hNK cells staining: After euthanasia, tumors were removed from the mice and mechanically dissociated into pieces as small as possible. The pieces were incubated in the presence of collagenase IV and DNAse I at 37 °C for 1 h, and strained through a 70 μm filter to get a single-cell suspension. Then the suspension was washed with PBS and used for subsequent staining. After staining with FVD, the cells were incubated with Fc Receptor Blocking Solution (BioLegend) for 10 min at room temperature. Then the cells were stained with fluorescence antibodies for 30 min at 4 °C, washed twice and resuspended with PBS, and finally analyzed by the flow cytometer. The following antibodies purchased from BioLegend were used in the staining: Alexa Fluor® 660 anti-human CD45 antibody, APC/Cyanine7 anti-human CD3 antibody, PE anti-human CD56 antibody.

MitoSox, LiperFluo and FerroOrange staining: The MitoSox™ Red was purchased from Invitrogen™. LiperFluo and FerroOrange were purchased from DOJINDO LABORATORISE. The collected cells were washed with pre-warmed PBS and treated with the probes under different conditions as follows: MitoSox™ Red, 5 μm in PBS, incubation at 37 °C for 10 min; LiperFluo and FerroOrange, 1 μm in PBS, incubation at 37 °C for 30 min. After that, the cells were washed and resuspended with PBS for analysis on the flow cytometer or subsequent staining.

IFN-γ, perforin and granzyme B staining: NK-92 cells were stimulated with 1 × Cell Stimulation Cocktail (plus protein transport inhibitors) (eBioscience™) for 8 h and stained with the Fixation / Permeabilization Solution Kit (BD Biosciences) following the manufacture’s instructions.

Flow cytometric data was analyzed with the FlowJo software (version 10.6.2, Treestar).

### Human PBMCs isolation

Ten milliliter peripheral blood were collected from healthy donors and diluted by 20 ml sterile PBS. We slowly added the diluted blood above 15 ml Ficoll (GE Healthcare) level in a 50 ml tube and centrifuged at 1500 rpm for 30 min without break-off. Then we collected PBMCs at the white middle interlayer and washed them with PBS twice for further use. The study was approved by the Medical Ethics Committee of Chinese PLA General Hospital (#20190315).

### Humanized mice and cell derived xenograft (CDX) tumor model preparation

The hIL-15 NOG mice were injected *i.v.* with 1 × 10^7^ human PBMCs. Seven days later, we carefully collected blood from submandibular vein of the mice and detected the proportion of human CD45^+^ (hCD45) cells to total lymphocytes in blood. The mice with more than 25% hCD45^+^ cells in total lymphocytes were selected and inoculated with 1 × 10^6^ GC cells *s.c.*. The tumor volume was measured using a caliper and calculated as 0.5 × length × width× width.

### Western blotting

The primary antibodies for IDO, AHR, β-actin were purchased from CellSignaling Technology and CYP1A1 was purchased from Santa Cruz Biotechnology. The detailed process has been described in our previous work [20].

### Genetic modification of the cell lines

To knock out IDO in SGC-7901 cells (SGC-7901^IDO-KO^), we constructed a LentiCRISPRV2 plasmid containing the sgRNA sequence: F: 5′- CACCGGGACAATCAGTAAAGAGTAC-3; R: 5′- AAACGTACTC TTTACTGATTGTCC-3′. Then the plasmid was co-transfected into HEK293T cells with the packaging plasmids pVSVg and psPAX2 to generated lentivirus. After infection and screening, the knocking out efficiency of IDO was proved by western blot. The plasmid without sgRNA sequence, which didn’t target at any genes, was used to construct the control SGC-7901 cell lines (SGC-7901^Con^).

To over-express GPX4 in NK-92 cells, we created a lentiviral overexpression vector expressing human GPX4: LV-EFS > Human GPX4 [NC_000019.10]-CMV > EGFP/T2A/Puro. The control vector was used as follows: LV-CMV > EGFP/T2A/Puro. After lentiviral package, NK-92 cells were transfected with the help of Lentiviral Transduction Enhancer (WZ Biosciences Inc.) following the manufacturer’s instructions. The EGFP^+^ cells were sorted, expanded and tested for GPX4 over-expression.

### NK cell killing assays

The target cells (K562 or SGC-7901) were labeled with 20 μM Calcein AM (BD Pharmingen) at 37 °C for 30 min. Then 7500 labeled target cells were co-cultured with NK-92 cells with the E:T ratio = 1:1 in a 96-well V-bottom plate for 6 h. The release of Calcein AM was measured using a fluorescence detector with 495 nm excitation wavelength and 520 nm emission wavelength. Three duplicate wells were set. The lysis efficiency was calculated with the formula: (experimental release- spontaneous release) / (maximum release-spontaneous release) × 100%. Spontaneous release: the Calcein AM from labeled cells at normal condition and maximum release: the Calcein AM from labeled cells with 1% Triton X-100 lysis buffer.

### Migration assays

The migration assays were performed in 24-well Transwell chambers with 8 μM pore polycarbonate membranes (Corning). 2 × 10^5^ NK-92^GPX4-con^ or NK-92^GPX4-high^ cells in 200 μL serum-free NK-92 culture medium were seeded into the upper chambers and 600 μL complete NK-92 medium containing 50 ng / ml chemokines CXCL12 and CCL21 were seeded into the bottom chambers. Then the system was incubated at 37 °C for 48 h and the cells penetrating to the lower side of membranes were fixed with methanol and stained with 0.1% crystal violet solution. The cell number was counted in microscopic field and the migration rate was calculated as followed: cell number in the bottom chambers / total cell number × 100%.

### Statistical analyses

All the results were demonstrated as mean ± SEM. The GraphPad Prism 8 software was used to carry out statistical analyses. For comparisons between two groups, the two-tailed unpaired t-test was used; for comparisons between one control with several treatment groups, one-way ANOVA with Dunnett’s post hoc test was used; for tumor volume comparisions, two-way ANOVA with Dunnett’s post hoc test was used. *P* value < 0.05 were considered statistically significant. The significance levels were defined as: * *p* < 0.05, ** *p* < 0.01, *** *p* < 0.001.

## Result

### IDO-produced L-KYN from GC cells impaired NK cells viability in vitro

In order to explore the effects of IDO-generated L-KYN on NK cells in the GC TME, we selected the GC cell line SGC-7901, which is reported to express abundant IDO [21], to construct an in vitro co-culture system with the human NK cell line NK-92 cells. Meanwhile, we set the normal gastric epithelial cell line GES-1 cells and GC cell line MGC-803, which barely express IDO [21], as the control. Firstly, we confirmed that the generation rate of L-KYN was significantly higher in the culture supernatant of SGC-7901 cells than GES-1 and MGC-803 cells (Fig. 1A), however those cells have no difference in L-KYN consuming rate (Fig. S1A). Then we built a non-contact system to co-culture NK-92 cells with SGC-7901, GES-1 or MGC-803 cells respectively for 24 or 48 hours. We found the remaining cell number of NK-92 was obviously decreased when co-cultured with SGC-7901 cells, compared with the negative control or GES-1 and MGC-803 cells (Fig. 1B). Consistently, the proportion of dead NK-92 cells, marked by FVD [22], increased substantially in the SGC-7901 co-culture system (Fig. 1C). We also examined whether the co-culture system would influence the proliferative capacity of NK cells. We detected the expression of ki-67, which was considered as a cell proliferation marker [23], in the viable (the FVD^—^subpopulations) NK-92 cells, and found no difference between those co-culture systems (Fig. S1B). Moreover, we also treated NK-92 cells with the culture supernatant of GES-1, MGC-803 or SGC-7901 cells, and the results showed that the supernatant from SGC-7901 also induced obvious cell death (Fig. 1D). In addition, the results above also demonstrated that there was no difference between GES-1 and MGC-803 cells, which could exclude other factors besides L-KYN between GC cells and normal gastric epithelial cell in inducing NK cell death. Then, to directly confirm the influence of L-KYN on NK cells’ viability, we stimulated NK-92 cells with L-KYN at the indicated concentrations for 24 or 48 h, and found obvious cell death related with the dose of L-KYN (Fig. 1E).

To further verify that IDO in gastric cancer cells played a vital role in promoting L-KYN-induced NK cell death in TME, we specifically knocked out IDO in SGC-7901 cells (SGC-7901^IDO-KO^) (Fig. S1C). ELISA assays were performed to confirm the obvious reduction of L-KYN in the supernatant (Fig. S1D). Then we co-cultured NK-92 cells with SGC-7901^IDO-KO^ for 48 h, and found the proportion of dead NK cells were significantly decreased, compared with the SGC-7901^Con^ cells (Fig. 1F). Consistently, the culture supernatant of SGC-7901^IDO-KO^ cells also showed less impact on NK-92 cells’ viability (Fig. 1G). However, if we additionally added L-KYN into the supernatant of SGC-7901^IDO-KO^ cells, the proportion of dead NK cells reversely increased (Fig. 1G).

Next, we isolated primary human NK cells (hNK) from healthy donors and also found L-KYN could induce obvious cell death (Fig. 1H). Besides, we treated primary hNK cells with the culture supernatant of GES-1, SGC-7901^Con^ or SGC-7901^IDO-KO^ cells respectively by the same methods above, and also found the supernatant of SGC-7901^Con^ led to more intensified hNK cell death compared with the GES-1 or SGC-7901^IDO-KO^ (Fig. 1I). And as above, the additionally added L-KYN promoted more hNK cell death in the GES-1 or SGC-7901^IDO-KO^ group (Fig. 1I).

### GC cells-generated L-KYN leads to NK cell death in the TME in vivo

**Fig. 2:**
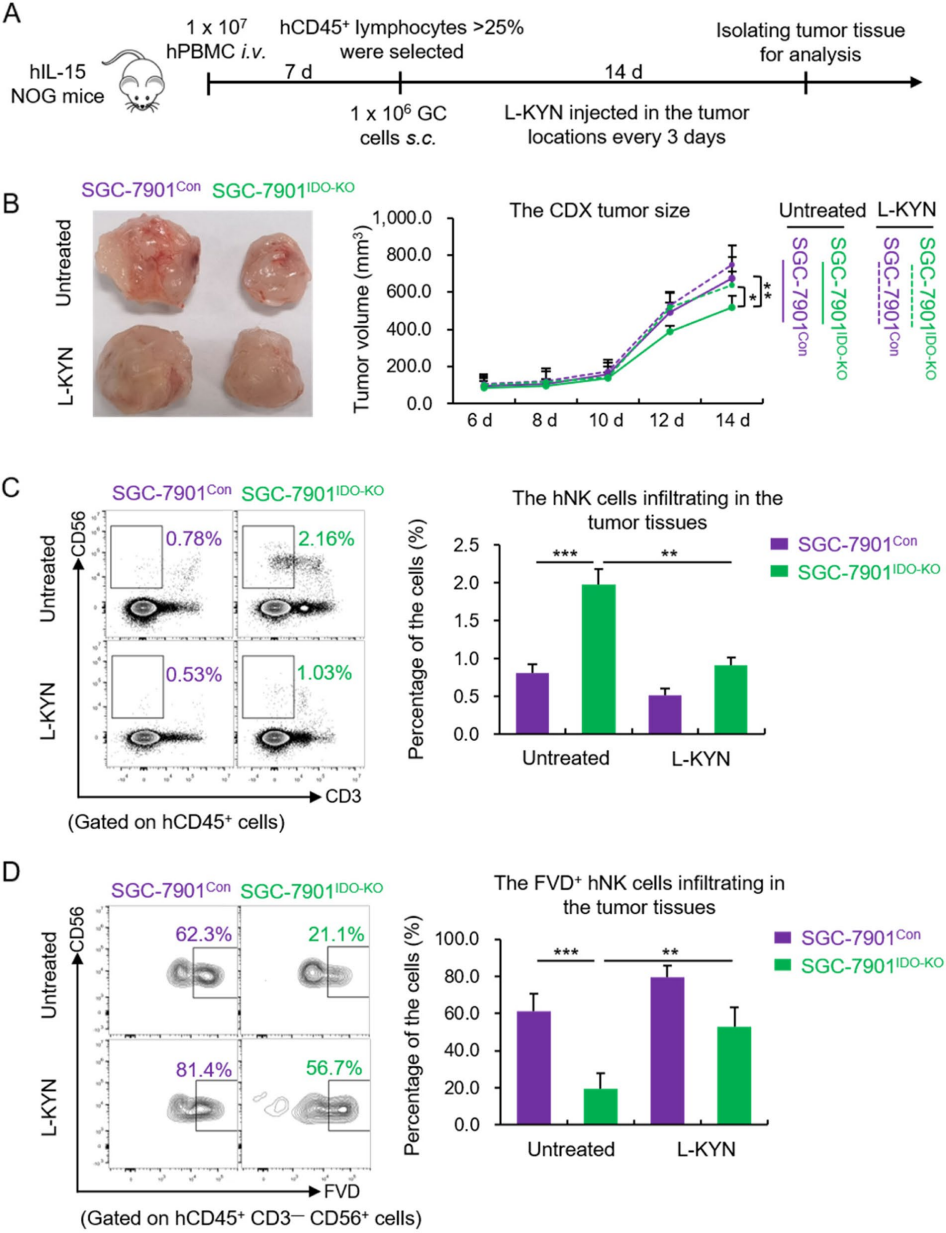
GC cells-generated L-KYN leads to NK cell death in the TME in vivo. **A** The schematic diagram to show the construction process of humanized CDX tumor model in hIL-15 NOG mice. **B** The size of CDX tumors from the mice of different groups, measured by a caliper at various time points. The pictures on the left show the harvested tumors in one representative experiment. **C** The flow cytometric results to show the percentage of the transferred hNK cells (defined as CD3^−^CD56^+^) in hCD45^+^ lymphocytes, which infiltrated in the CDX tumor tissues with or without L-KYN treatment injected in the paratumor sites. The pictures on the left side were representative results. **D** The proportion of FVD^+^ hNK cells infiltrated in the tumor tissues, detected by flow cytometry. The results were replicated in 3 independent experiments with *n* = 4 for each group (**B - D**). * *p* < 0.05, ** *p* < 0.01, *** *p* < 0.001

Next, we constructed a cell derived xenograft (CDX) tumor model based on humanized-PBMC (hu-PBMC) mice [24]. 1 × 10^7^ healthy donor-derived PBMCs were injected *i.v.* into the hIL-15 NOG mice. Seven days later, we selected the successfully built hu-PBMC mice and seeded SGC-7901^Con^ or SGC-7901^IDO-KO^ cells *s.c.* into the mice. After 14 days, the tumor tissues were collected and the infiltrating human NK (hNK) cells were analyzed by flow cytometry (Fig. 2A). We found the size of SGC-7901^IDO-KO^-formed tumors was smaller than that of the SGC-7901^Con^-formed (Fig. 2B). Besides, the L-KYN concentration in the dissociation supernatant of SGC-7901^IDO-KO^ cells - formed CDX tumors was reduced (Fig. S2A). However, it was only slightly influenced in the host serum of different groups (Fig. S2B). The flow cytometric results showed that the total proportion of hNK in SGC-7901^IDO-KO^-formed tumor was significantly higher than that in the SGC-7901^Con^-formed (Fig. 2C). In consistence, the percentage of dead hNK cells was obviously lower in the former than the later (Fig. 2D). Meanwhile, we additionally injected 4 μg L-KYN *s.c.* into the paratumor tissues every other day and found L-KYN apparently decreased the infiltration (Fig. 2C), while raised the death rate (Fig. 2D) of hNK cells in the tumor.

In all, based on the in vitro and in vivo evidence above, we proved that L-KYN produced by IDO in gastric cancer cells was involved in shaping the TME that led to NK cell death.

### L-KYN introduced NK cell ferroptosis in an AHR-independent way

**Fig. 3:**
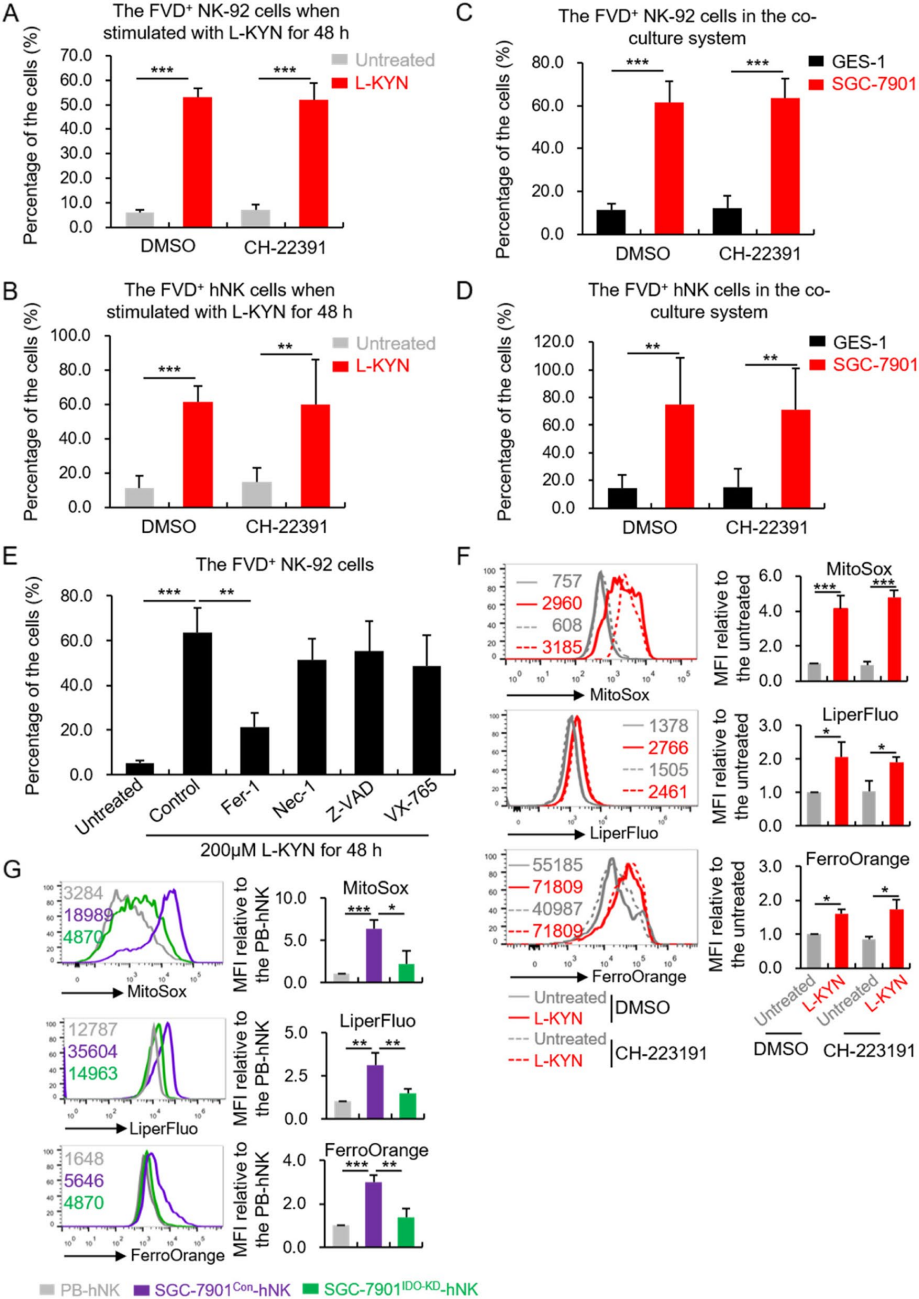
L-KYN induced NK cell ferroptosis in an AHR-independent way. **A - D** The proportion of FVD^+^ NK-92 or hNK cells after being treated with 200 μm L-KYN (**A** and **B**) or co-cultured with GES-1 or SGC-7901 cells (**C** and **D**) at the presence of the AHR inhibitor CH-223191 (1 μm) for 48 h, detected by flow cytometry. **E** The proportion of FVD^+^ NK-92 cells when treated with 200 μm L-KYN for 48 h, combining with 2 μm Fer-1, 1 μm Nec-1, 10 μm Z-VAD or 1 μm VX-765 respectively, detected by flow cytometry. **F** The flow cytometric detection for the mean fluorescence intensity (MFI) of MitoSox, LiperFluo and FerroOrange in NK-92 cells when treated with 200 μm L-KYN combined with or without 1 μm CH-223191 for 48 h. The pictures on the left were representative results, on which the numbers indicated the MFI of different samples. The MFI was normalized to the untreated + DMSO group. **G** The levels of MitoSox, LiperFluo and FerroOrange in primary hNK cells (hCD45^+^CD3^−^CD56^+^) infiltrating in the tumor tissues formed by SGC-7901^Con^ or SGC-7901^IDO-KO^ cells in humanized mice, analyzed by flow cytometry. The MFI in the tumor-infiltrated hNK cells was normalized to that in the peripheral blood hNK cells (PB-hNK) of the same mice. All the results were replicated in 3 (**B, D, G**) or 4 (**A, C, E, F**) independent experiments. * *p* < 0.05, ** *p* < 0.01, *** *p* < 0.001

It has been reported that L-KYN is an efficient AHR agonist in vivo [10]. To investigate whether the L-KYN induced-NK cell death was associated with AHR pathway, we treated NK-92 cells with L-KYN in combination with the AHR antagonist CH-223191 (1 μM). We affirmed that CH-223191 effectively blocked AHR activation by L-KYN in NK-92 or hNK cells through detecting the expression of CYP1A1 (Fig. S3A and B), which is generally considered as a surrogate for the activity of AHR pathway [25]. Meanwhile, we found inhibition on AHR activity showed no influence on L-KYN-induced NK-92 or hNK cells death (Fig. 3A and B). We observed the same results in the co-culture system with GES-1 or SGC-7901 cells at the presence of CH-223191 (Fig. 3C and D). Those evidence revealed that L-KYN-induced NK cell death was not associated with AHR pathway.

To clarify how L-KYN triggered NK cell death, we supplemented the specific inhibitors for different types of cell death into the L-KYN stimulation assays, including the ferroptosis inhibitor – ferrostain-1 (Fer-1, 2 μm), necrosis inhibitor – necrostatin-1 (Nec–1, 1 μm), apoptosis inhibitor – Z-VAD-fmk (Z-VAD, 10 μm) [26] and pyroptosis inhibitor – VX-765 (1 μm). It turned out the treatment of Fer-1 showed the most significant restraining on L-KYN – induced NK cell death (Fig. 3E), which meant ferroptosis might play a major role in the process. It has been reported that ferroptosis can be featured by the accumulation of cellular ROS, lipid peroxides and iron [27]. Hence, we measured them with different fluorescence probes (MitoSox for ROS, LiperFluo for lipid peroxides, FerroOrange for iron) by flow cytometry and found L-KYN stimulation indeed led to higher levels of these indicators in NK-92 cells (Fig. 3F), which suggested an increased ferroptosis level. In addition, blocking AHR pathway by CH-223191 still showed no influence on the changes of ferroptosis (Fig. 3F). We further detected those indicators in hNK cells from the humanized CDX tumor model. Compared with the hNK cells in peripheral blood and SGC-7901^IDO-KO^ – formed tumor tissues, those infiltrating in the SGC-7901^Con^ – formed tumor exhibited significantly higher level of ROS, lipid peroxides and iron accumulation (Fig. 3G), which also suggested a higher level of ferroptosis.

### hNK cells with higher GPX4 expression showed resistance to L-KYN induced ferroptosis

**Fig. 4:**
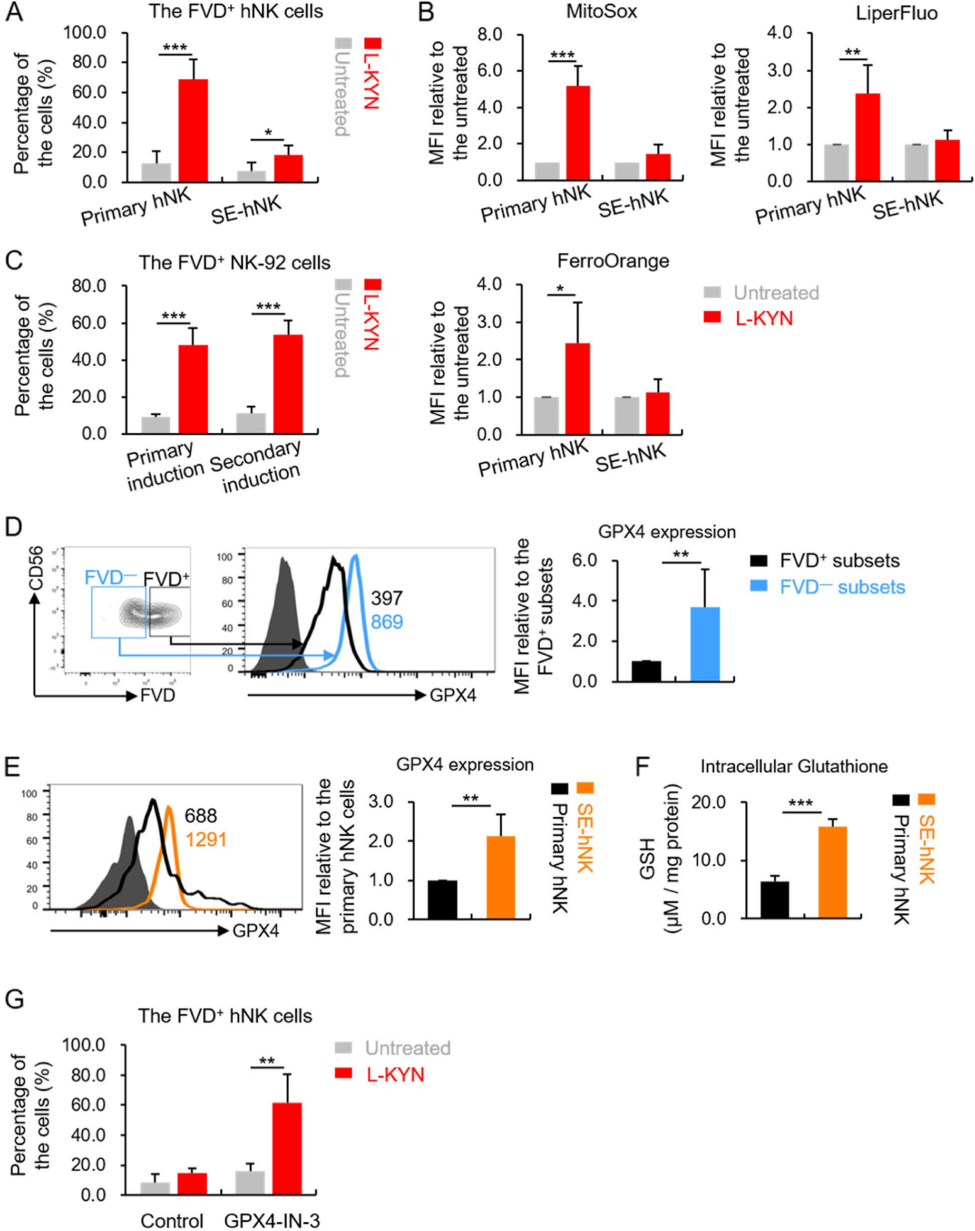
The hNK cells with higher GPX4 expression showed resistance to L-KYN induced ferroptosis. **A** The percentage of FVD^+^ freshly isolated hNK cells (primary hNK cells) after being stimulated with 200 μm L-KYN for 48 h in vitro, detected by flow cytometry (the left two columns of the diagram). Then the survival hNK cells were expanded in vitro (SE-hNK cells) and stimulated with L-KYN again. The proportion of FVD^+^ cells was also detected by flow cytometry (the right two columns of the diagram). **B** and **C** The flow cytometric analysis for the MFI of MitoSox, LiperFluo and FerroOrange in the primary and SE- hNK cells (**B**), or the proportion of FVD^+^ NK-92 cells (**C**) as in Fig. 4A. **D** The expression of GPX4 in the FVD^−^ or FVD^+^ primary hNK cells when stimulated with L-KYN for 48 h, analyzed by flow cytometric. The pictures on the left were representative flow cytometric results. **E** The expression of GPX4 in the SE-hNK cells compared with the primary hNK cells, detected with flow cytometry. **F** The intracellular GSH level measured in the whole cell lysates of primary or SE- hNK cells. **G** The SE-hNK cells were stimulated with or without L-KYN at the presence of GPX4-IN-3 for 48 h. Then the percentage of FVD^+^ cells was measured by flow cytometry. All the results were replicated in 3 (**A, B, D, E, F, G**) or 4 (**C**) independent experiments. * *p* < 0.05, ** *p* < 0.01, *** *p* < 0.001

When induced primary hNK cells death with L-KYN, we found an interesting phenomenon: if we expanded the surviving hNK cells after L-KYN treatment (named as SE-hNK cell in this article, meant surviving and then expanded human NK cells) and stimulated them with L-KYN again, the cell death was only slightly increased compared with the untreated (Fig. 4A), and the changes of ROS, lipid peroxides and iron content was consistent with that (Fig. 4B). The evidence suggested that the SE-hNK cells were somewhat resistant to L-KYN retreatment-induced ferroptosis. Interestingly, this phenomenon could only be observed in primary hNK cells. When we expanded the NK-92 cells that survived from the initial treatment of L-KYN, the proportion of dead cells was still substantially increased with a second stimulation (Fig. 4C). This might be attributed to the heterogeneity of primary hNK cells and homogeneity of the NK cell lines [28].

A large number of studies have reported that glutathione peroxidase-4 (GPX4) in cells can reduce the level of lipid peroxides through glutathione (GSH) oxidation pathway, thereby inhibiting ferroptosis [29, 30]. Therefore, some cells with abundance GPX4 are resistant to ferroptosis. For example, Eduardo P. Amaral *et. al.* found some macrophages expressed more GPX4 in cells, contributing to their survival from the *Mycobacterium tuberculosis* infection-induced ferroptosis [22]. Through flow cytometry, we found the expression level of GPX4 in FVD^−^ primary hNK cells was obviously higher than that in the FVD^+^ subsets when treated with L-KYN (Fig. 4D). In addition, the mean GPX4 expression level as well as the cellular GSH content were also higher in the SE-hNK cells compared with the primary cells (Fig. 4E and F). Furthermore, if we supplemented the specific inhibitor of GPX4 (GPX4-IN-3, 1 μm) when treated the SE-hNK cells with L-KYN, the cell death was significantly increased again (Fig. 4G). Based on the evidence above, we considered that due to the heterogeneity of primary hNK cells [28], there existed a group of NK cells, in which the expression level of GPX4 was higher than the others. These NK cells showed resistance to L-KYN-induced ferroptosis.

### The GPX4-overexpressed NK-92 cells showed therapeutic potential towards GC

**Fig. 5:**
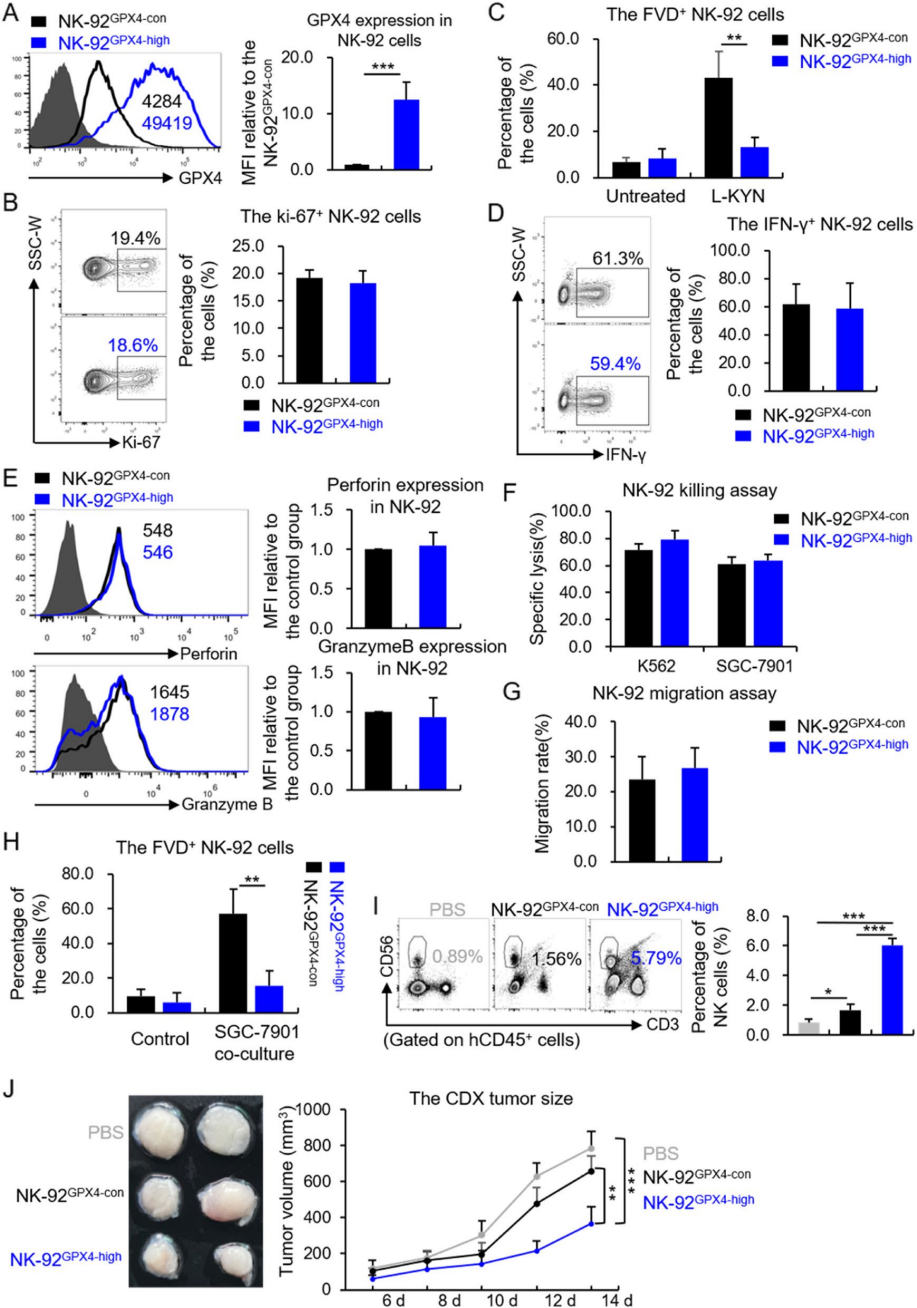
The GPX4-overexpressed NK-92 cells showed therapeutic potential towards GC. **A** The expression of GPX4 in NK-92^GPX4-high^ or NK-92^GPX4-con^ cells, measured by flow cymetry. The pictures on the left were representative results. **B** The percentage of ki-67^+^ subsets in NK-92^GPX4-high^ or NK-92^GPX4-con^ cells under normal conditions. **C** The percentage of FVD^+^ NK-92^GPX4-high^ or NK-92^GPX4-con^ cells when they were treated with or without L-KYN for 48 h, detected by flow cytometry. **D** and **E** The expression of IFN-γ(D), perforin and granzyme B (E) in NK-92^GPX4-high^ or NK-92^GPX4-con^ cells when stimulated with the cell stimulation cocktail (plus protein transport inhibitors) for 8 h. **F** The efficiency of NK-92^GPX4-high^ or NK-92^GPX4-con^ cells killing target cells when they were co-cultured for 6 h at an E: T ration of 1: 1. **G** The migration rate of NK-92^GPX4-high^ or NK-92^GPX4-con^ cells, measured by transwell assays. **H** The percentage of FVD^+^ NK-92^GPX4-high^ or NK-92^GPX4-con^ cells when they were co-cultured with or without SGC-7901 for 48 h. **I** The proportion of NK cells infiltrated in the SGC-7901-formed solid tumors in humanized hIL-15 NOG mice, after the mice being treated with PBS, 1 × 10^7^ NK-92^GPX4-high^ or NK-92^GPX4-con^ cells. **J** The CDX tumors size, measured by a caliper at various time points. The pictures on the left were presentative results. All the results were replicated in 2 (**I** and **J**, *n* = 4 in per group) or 3 (**A - H**) independent experiments. * *p* < 0.05, ** *p* < 0.01, *** *p* < 0.001

NK-92 cell is frequently used as an editable platform for developing NK cells-based immunotherapeutic products [31, 32]. As the hNK cells with higher GPX4 expression were tolerant to L-KYN-induced cell loss in TME, we thought this strategy might be used to develop a NK-92 based immunotherapy towards GC. Thus, we constructed GPX4-overexpressed NK-92 cells (termed as NK-92^GPX4-high^ cells, the WT control was termed as NK92^GPX4-con^), and confirmed it with flow cytometry (Fig. 5A). Firstly, we detected whether GPX4 over-expression would affect the homeostasis of NK-92 cells by examining Ki-67 expression, a surrogate for cell proliferative rate, and the death rate under normal conditions. The results showed there were no difference between these two groups on percentages of Ki-67^+^ (Fig. 5B) or FVD^+^ cells (Fig. 5C, the untreated group). Moreover, we tested whether GPX4 over-expression would have influence on NK-92 effector functions. We found the secretion of IFN-γ (Fig. 5D), the expression of perforin or granzyme B (Fig. 5E), the direct killing ability towards target cells (K562 and SGC-7901 cells) (Fig. 5F) as well as the migration rate (Fig. 5G) all showed no difference between NK-92^GPX4-con^ and NK-92^GPX4-high^ cells. Then, through in vitro assays, we proved that NK-92^GPX4-high^ cells were resistant to cell death induced by L-KYN treatment (Fig. 5C) or the co-culture with SGC-7901 cells (Fig. 5H). Based on this, we treated the humanized CDX tumor mice with PBS (set as negative control), 1 × 10^7^ NK-92^GPX4-con^ or NK-92^GPX4-high^ cells through tail vein. It turned out that there were much more NK cells infiltrated in the tumor tissues from the NK-92^GPX4-high^ treated group than the other two groups (Fig. 5I). Consistently, the development of tumor was also apparently inhibited by NK-92^GPX4-high^ cells compared with the negative control or NK-92^GPX4-con^ cells (Fig. 5J).

## Discussion

**Fig. 6:**
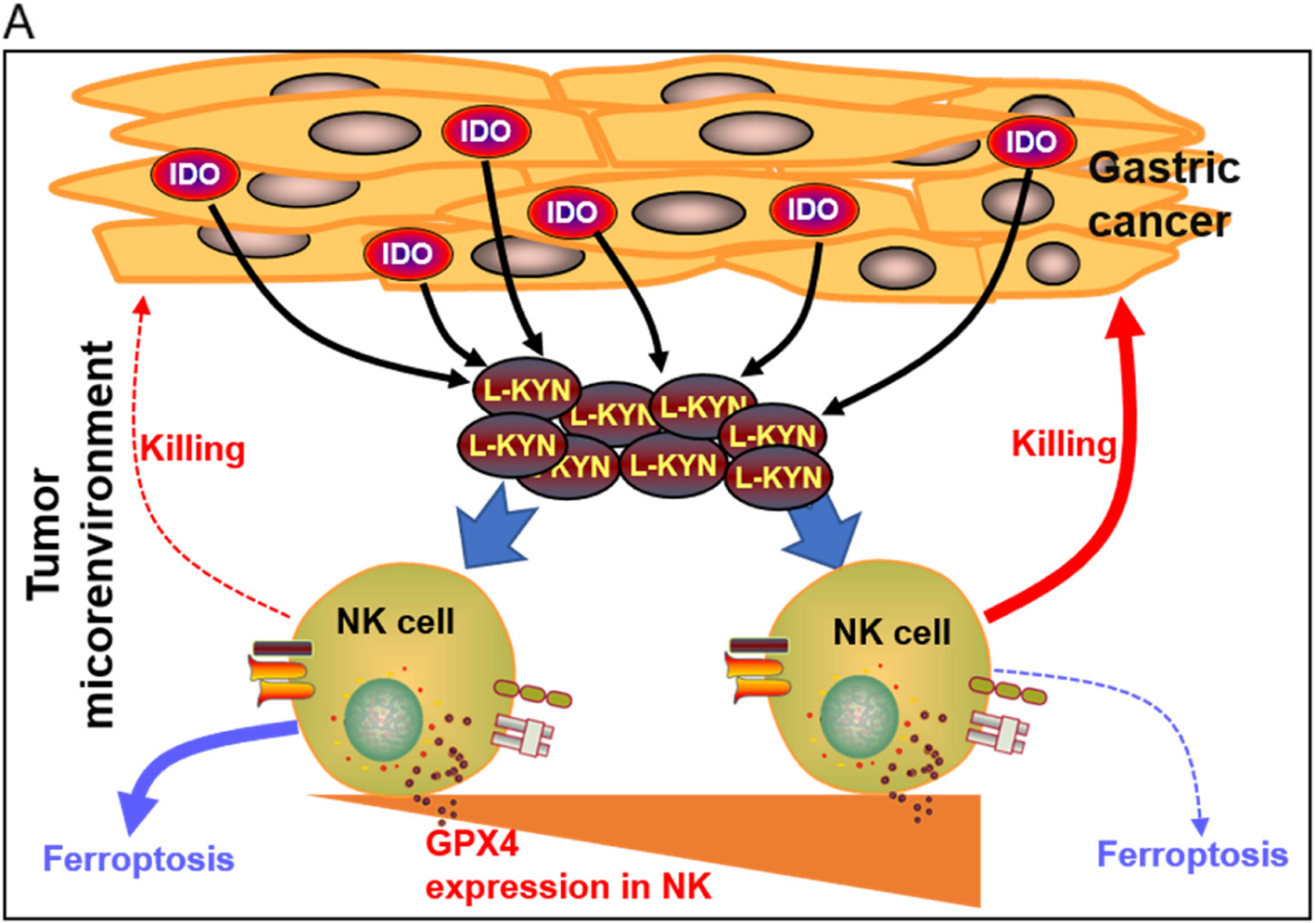
The schematic diagram to show L-KYN from GC cells leads to NK cell ferroptosis. A L-KYN was metabolized by IDO in GC cells and released into the TME. The NK cells with lower GPX4 expression were sensitive to L-KYN induced ferroptosis, reducing the killing efficiency towards GC cells. This could lead to immune escape of GC cells from the surveillance and clearance of NK cells. However, some NK cells with higher GPX4 expression could survive the L-KYN stimulation and contribute to anti-tumor responses

Compared with T cells, NK cells have unique advantages in tumor immunotherapy: 1. their survival time in vivo is relatively short, which is related with the lower unpredictable risk; 2. due to the intrinsic receptors on cell surface, they can directly recognize and kill cancerous cells without sensitization; 3. the allogeneic NK cells-based immunotherapy hasn’t seen severe graft-versus-host reaction [7]. Therefore, NK cell-based immunotherapy may become a breakthrough point for tumor treatment. In fact, the adoptive NK transfer has achieved exciting effects in the treatment of hematological malignancies, but it has not shown obvious efficacy in the clinical experiments of GC [33]. The main reason for the low response rate in solid tumors is believed to be related with the immunosuppressive effects of the TME [34], however the mechanisms involved are still misty. In the present study, we uncovered that the IDO-generated L-KYN from GC cells triggered ferroptosis in NK cells, which was one major reason leading to NK cells loss in the TME (Fig. 6). Although previous studies have reported L-KYN could inhibit NK cytotoxicity [35, 36], it has to be noted that the phenomenon is only observed when NK cells are continuously activated with excessive IL-2 [35] or the FC21 feeder cells [36], which are inconsistent with what are actually happening in the TME.

Recently, ferroptosis has been recognized as a form of iron-dependent cell death, which is implicated in multiple pathological process, including neurodegenerative diseases, acute renal failure, heart ischemia/reperfusion injury and tumorigenesis [37]. Xingzhe Ma *et. al.* reported that TME induced ferroptosis in intra-tumoral CD8^+^ T cells, leading to impaired antitumor abilities [38] . However, ferroptosis has rarely been studied in NK cells before us.

As for how L-KYN induced NK cell ferroptosis, we initially thought it might because L-KYN was an effective agonist of AHR pathway. The AHR activation was reported to trigger oxidative stress in NK [36], which could induce ferroptosis. However, supplementing CH-223191, a generally used AHR antagonist, showed no influence on L-KYN induced NK ferroptosis. This suggested an AHR independent way in which L-KYN might play a role. We will uncover the mechanisms in depth in the subsequent work.

## Conclusion

Increasing the NK cell number or improving their viability has become one of the main strategies for NK-based immunotherapy. By accident, we found some primary hNK cells with abundant GPX4 showed resistance to L-KYN or GC TME induced ferroptosis (Fig. 6). And inspired by this, we constructed the NK-92^GPX4-high^ cells and found they showed significantly curative effects in the humanized mice with CDX tumor. In all, our work might provide new strategy to develop NK-based immunotherapy for GC treatment in clinical.

## Supplementary Information

**Additional file 1: Supplementary Fig. 1.** A. The remaining concentration of L-KYN in different culture medium of GES-1, MGC-803 or SGC-7901 cells at the indicated time points, measured by ELISA. 200 μM L-KYN was supplemented into the medium at the initial time point. B. The proportion of ki-67^+^ cell subsets in the FVD^+^ or FVD^−^ NK-92 cells, detected by flow cytometry. C. The western blot result to show the knocking out efficiency of IDO in SGC-7901 cells. β-actin was served as the internal reference. D. The concentration of L-KYN in the culture supernatant of the indicated SGC-7901 cells, measured by ELISA at different time points. The results were replicated in 3 independent experiments. ** p < 0.01, *** p < 0.001. **Supplementary Fig. 2.** A and B. The L-KYN concentration in the dissociation supernatant of SGC-7901^Con^ or SGC-7901^IDO-KO^ cells - formed CDX tumors (A) or the host serum (B), measured by ELISA. The results were replicated in 3 independent experiments with n = 4 for each group. * p < 0.05, ** p < 0.01, *** p < 0.001. **Supplementary Fig. 3.** A and B. The western blot results to show the protein expression level of AHR and CYP1A1 in NK-92 cells (A) or hNK cells (B) when treated with 1 μM CH-223191 for 48 h. β-actin was served as the internal reference.

## Data Availability

The authors declare that all data supporting the fndings of this study are available in the main text and supplementary materials. Any other relevant data are available from the corresponding author upon reasonable requests.

## Abbreviations

GC: Gastric cancer
TME: Tumor microenvironment
NK cell: natural killer cell
L-KYN: L-kynurenine
IDO: Indoleamine 2,3-dioxygenase
DC: Dendritic cell
AHR: Aryl hydrocarbon receptor
GPX4: Glutathione Peroxidase 4
PB-hNK: Peripheral blood-derived human NK
Fer-1: Ferrostatin-1
Nec-1: Necrostatin-1
Z-VAD: Z-VAD-FMK
FVD: Fixable Viability Dye
CDX: Cell derived xenograft
SE-hNK: Surviving and then expanded human NK
