# Supplementary material for: L-kynurenine induces NK cell loss in gastric cancer microenvironment via promoting ferroptosis
Source: J Exp Clin Cancer Res. 2023 Mar 1;42:52. doi: 10.1186/s13046-023-02629-w (PMC9976385; doi:10.1186/s13046-023-02629-w)
Supplement: Supplementary file 1 — Additional file 1: Supplementary Fig. 1. A. The remaining concentration of L-KYN in different culture medium of GES-1, MGC-803 or SGC-7901 cells at the indicated time points, measured by ELISA. 200 μM L-KYN was supplemented into the medium at the initial time point. B. The proportion of ki-67+ cell subsets in the FVD+ or FVD− NK-92 cells, detected by flow cytometry. C. The western blot result to show the knocking out efficiency of IDO in SGC-7901 cells. β-actin was served as the internal reference. D. The concentration of L-KYN in the culture supernatant of the indicated SGC-7901 cells, measured by ELISA at different time points. The results were replicated in 3 independent experiments. ** p < 0.01, *** p < 0.001. Supplementary Fig. 2. A and B. The L-KYN concentration in the dissociation supernatant of SGC-7901Con or SGC-7901IDO-KO cells - formed CDX tumors (A) or the host serum (B), measured by ELISA. The results were replicated in 3 independent experiments with n = 4 for each group. * p < 0.05, ** p < 0.01, *** p < 0.001. Supplementary Fig. 3. A and B. The western blot results to show the protein expression level of AHR and CYP1A1 in NK-92 cells (A) or hNK cells (B) when treated with 1 μM CH-223191 for 48 h. β-actin was served as the internal reference. [file 13046_2023_2629_MOESM1_ESM.docx]

**Supplementary material**


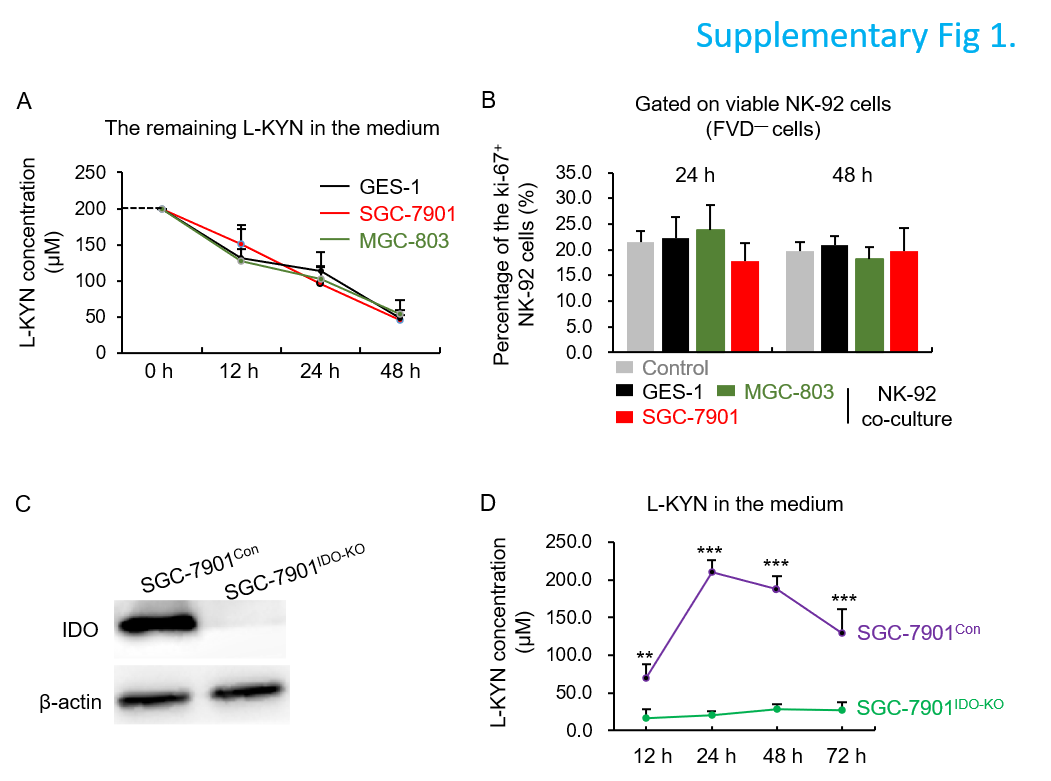
 **Supplementary Fig 1. A.** The remaining concentration of L-KYN in different culture medium of GES-1, MGC-803 or SGC-7901 cells at the indicated time points, measured by ELISA. 200 μM L-KYN was supplemented into the medium at the initial time point. **B.** The proportion of ki-67^+^ cell subsets in the FVD^+^ or FVD^-^ NK-92 cells, detected by flow cytometry. **C.** The western blot result to show the knocking out efficiency of IDO in SGC-7901 cells. β-actin was served as the internal reference. **D.** The concentration of L-KYN in the culture supernatant of the indicated SGC-7901 cells, measured by ELISA at different time points. The results were replicated in 3 independent experiments. ** p < 0.01, *** p < 0.001.


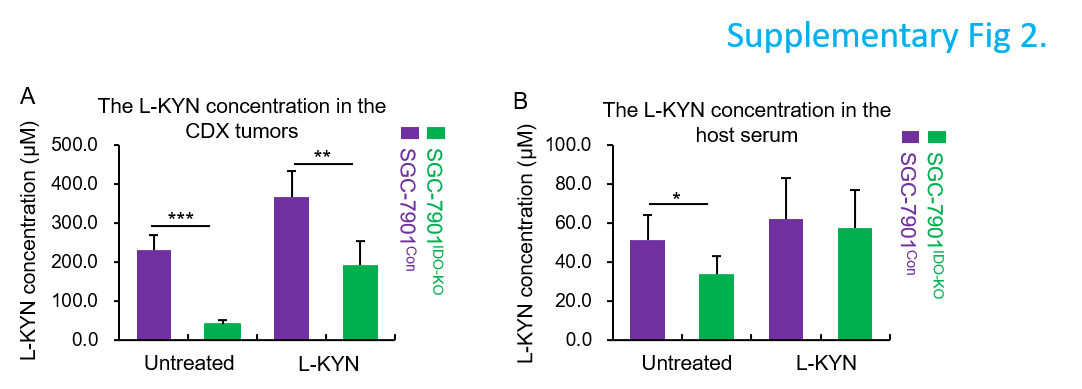
 **Supplementary Fig 2. A and B.** The L-KYN concentration in the dissociation supernatant of SGC-7901^Con^ or SGC-7901^IDO-KO^ cells - formed CDX tumors (A) or the host serum (B), measured by ELISA. The results were replicated in 3 independent experiments with n = 4 for each group. * p < 0.05, ** p < 0.01, *** p < 0.001.


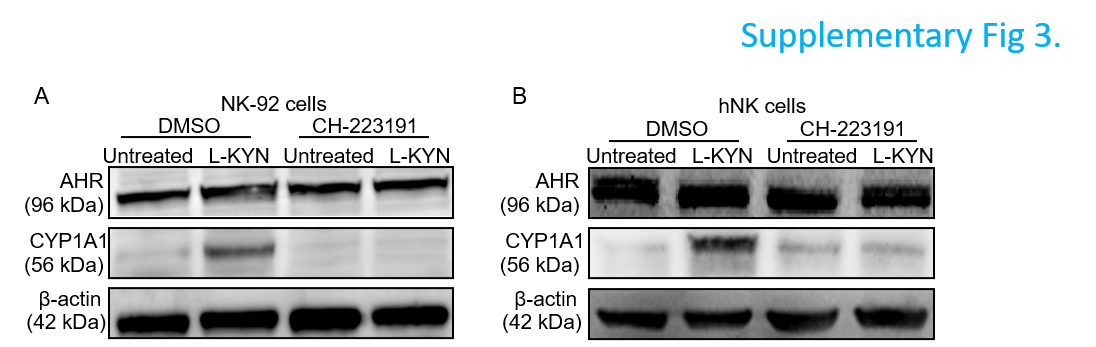
 **Supplementary Fig 3. A and B.** The western blot results to show the protein expression level of AHR and CYP1A1 in NK-92 cells (A) or hNK cells (B) when treated with 1 μM CH-223191 for 48 h. β-actin was served as the internal reference.
